# Tunable narrow-and-sharp defect modes and transmission peak degeneracy in periodic superconducting photonic crystals

**DOI:** 10.1371/journal.pone.0341241

**Published:** 2026-01-23

**Authors:** Aimei Liu, Han Gao, Yongjun Xiao, Junze Zheng, Quankun Zhang

**Affiliations:** 1 School of Physics and Electronic-Information Engineering, Hubei Engineering University, Xiaogan, Hubei Province, People’s Republic of China,; 2 School of Electric and Information Engineering, Hubei University of Science and Technology, Xianning, Hubei Province, People’s Republic of China; Beni-Suef University, EGYPT

## Abstract

The transmittance and the defect mode (DM) are investigated in a one-dimensional superconducting photonic crystal (PC). The structure is composed of periodically stacked SD/DS pairs with a strict central symmetry, where S and D denote superconductor and dielectric layers, respectively. A sharp and narrow DM emerges at the center of the photonic bandgap. Transmission peak degeneracy can be observed by increasing the period number. With this fractal-like behavior, the transmittance is highly sensitive to external stimuli. The DM wavelength *λ*_*c*_ linearly blue-shifts with the hydrostatic pressure, and monotonically red-shifts with the temperature. While increasing the incident angle, *λ*_*c*_ bule-shifts first smoothly and then sharply. Notably, enhanced tunability is achieved under low pressures, elevated temperatures, and large incidence angles, suggesting superior modulation capabilities. High pressure sensitivity of the DM wavelength, up to 128nm/GPa, is achieved with low deformations. Moreover, the DM maintains near-perfect transmittance unless the temperature is higher than the critical temperature of the superconductor. These findings highlight the potential of such defect-engineered superconducting PCs for high-performance optical sensing applications.

## 1. Introduction

Photonic crystals (PCs), with their ability to manipulate light propagation through engineered bandgaps (PBGs), have become a cornerstone of modern photonics, enabling unprecedented control over light for applications in waveguiding, lasing, and sensing [1–3]. A particularly powerful feature is the introduction of defect modes (DMs) by breaking the PC’s translational symmetry, which creates highly localized states within the PBG that function as sharp resonant transmission channels [4, 5]. A particularly powerful feature is the introduction of defect modes (DMs) by breaking the PC’s translational symmetry, which creates highly localized states within the PBG that function as sharp resonant transmission channels [6,7]. The dynamic tunability of DMs is a key area of research, often achieved by incorporating functional materials whose refractive indices respond to external stimuli. This has been demonstrated using semiconductors [8], liquid crystals [9], metamaterials [10], and piezoelectrics [11]. Analytical approaches have also been developed to precisely engineer DMs in multi-defect structures, providing a foundational understanding of their formation [12]. Analytical approaches have also been developed to precisely engineer DMs in multi-defect structures, providing a foundational understanding of their formation [13]. Among the various tunable materials, high-temperature superconductors (HTS) offer a unique and compelling combination of properties for advanced photonics, including negligible ohmic loss, low dispersion, and a strongly temperature-dependent electromagnetic response that is crucial for quantum computing [14] and multi-channel filtering [15]. The specific HTS material HgBa_2_Ca_2_Cu_3_O_8+δ_ (Hg-1223), chosen for this work, has been extensively studied for its defect chemistry and superior superconducting properties [16], making it an excellent candidate for photonic applications where material purity and response are critical. Its use in tunable PC structures, for instance in combination with GaAs, has been previously demonstrated [17], and studies have shown that the choice of superconductor (e.g., high-*T*_*c*_ versus low-*T*_*c*_) directly governs the spectral position of the defect mode [18].

Consequently, one-dimensional superconducting PCs have been extensively explored for creating tunable photonic devices. The DM can be modulated by temperature [19], defect layer thickness [20], and incident angle [21], and structures have been designed to generate complex resonance features like Fano lineshapes [22]. Recent studies have further enriched this field, demonstrating complex structures involving metamaterial-superconductor hybrids [23], multichannel filters in waveguide geometries [24], the integration of superconductors with liquid crystals [25] or graphene to explore novel effects like Goos-Hänchen shifts [26], and symmetric ternary designs [27]. Furthermore, the tunability of photonic bandgaps themselves via external stress has been demonstrated in other material systems, highlighting a broader pathway for dynamic control [28]. Despite these significant advances, a specific and highly promising configuration remains underexplored: a mirror-symmetric superconducting PC structure engineered to exhibit a distinct optical fractal phenomenon. While fractal and topological photonic effects are gaining traction for their novel control over light [29,30], their intersection with superconducting photonics is rare. Previous works on superconducting PCs have primarily focused on conventional (AB)^N^-type structures or complex hybrid designs. The combination of a strictly symmetric SD/DS architecture, a central dielectric defect, and the resulting transmission peak degeneracy—a distinct fractal-like behavior where side peaks merge rather than split as the period number increases—has not been reported. This unique phenomenon, when combined with the exceptional tunability of the Hg-1223 superconductor, promises a new pathway to ultra-sharp, highly sensitive resonances for precision sensing.

Our work addresses this gap by proposing a one-dimensional superconducting PC with strict mirror symmetry. The central two adjacent dielectric layers naturally form a defect. We demonstrate that this structure supports a remarkably sharp and narrow DM, which exhibits an unusual transmission peak degeneracy, an optical fractal-like behavior that enhances its sensitivity. We systematically investigate the DM’s response to hydrostatic pressure, temperature, and incident angle, revealing linear and monotonic tuning with high sensitivity. The unique synergy between the symmetric design, the peak degeneracy, and the superior electro-optical properties of HTS establishes a new paradigm for developing high-performance, multi-functional optical sensors. The remainder of this paper is structured as follows: Section 2 details the structure and the theoretical method, Section 3 presents the optical fractal-like properties and tuning characteristics, and Section 4 provides a comprehensive sensitivity analysis before concluding in Section 5.

## 2. Structure and method

**Fig 1:**
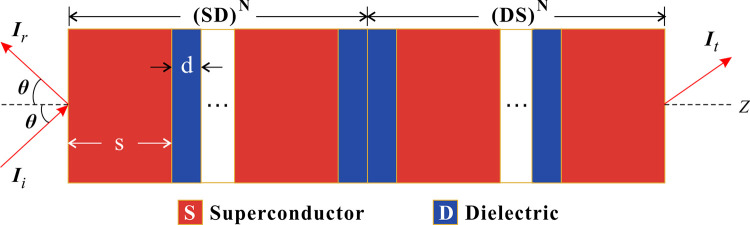
Scheme of the periodic photonic crystal.

The proposed one-dimensional photonic crystal (PC) consists of alternating superconducting (S) and dielectric (D) layers, where S is the superconductor HgBa_2_Ca_2_Cu_3_O_8+δ_ (Hg-1223) and D is the dielectric gallium arsenide (GaAs). Here, the superconductor is chosen specifically targeting enhanced tunability, lower loss, and sharper resonances compared to conventional materials [16,26]. These unique advantages of the superconductors makes superior performance in tunable and high signal-to-noise ratio (SNR) filter or sensor within a larger cryogenic system [14,22]. As illustrated in Fig 1, the PC exhibits strict mirror symmetry about its center, with the left half comprising N periodic (SD) bilayers, denoted as PC_*NL*_=(SD)^*N*^, and the right half as the inverted counterpart, PC_*NR*_=(DS)^*N*^, yielding the full structure PC_*N*_=(SD)^*N*^(DS)^*N*^. PC_*N*_ is surrounded by air. The practical fabrication of the proposed PC_*N*_ is considered feasible through advanced thin-film techniques such as Pulsed Laser Deposition (PLD) [31]. A key enabling factor would be the use of an appropriate epitaxial buffer layer deposited on the GaAs substrate. This buffer layer would serve to mitigate interdiffusion and promote the c-axis-oriented growth of the Hg-1223 film at a compatible substrate temperature.

For different period numbers, this complex photonic system can be truncated to a multilayer structure of finite length. At the center of the truncated PC, the two adjacent D layers serve as a defect, breaking translational symmetry and enabling defect-mode (DM) formation. The initial refractive indexes of S and D are *n*_*s*0_ = 0.9994 and *n*_*d*0_ = 3.5678, respectively [26,32], both sensitive to external stimuli such as temperature and pressure. Each layer is designed as a quarter-wave optical thickness *s,d* = *λ*_0_/[4Re(*n*_*s,d*_)] which depends on the refractive index. Here, we set *λ*_0_ = 1.55 μm as the center wavelength, yielding initial thicknesses *s*_0_ = 387.7 nm and *d*_0_ = 108.6 nm. This wavelength was selected for its strong technological alignment with the standard telecommunication C-band, granting access to a mature ecosystem of high-precision optical components (e.g., lasers, detectors) for characterization and future integration. From a practical standpoint, 1.55 μm represents an optimal balance where the required layer thicknesses are feasible for advanced deposition techniques like PLD, while simultaneously ensuring minimal material loss in both the superconducting and dielectric layers. Consequently, this choice allows us to fully leverage the quarter-wave interference principle to create strong photonic bandgaps and sharp defect modes, making it ideal for developing high-performance photonic sensors. The transverse magnetic (TM) polarized light *I*_*i*_ impinges at an angle *θ* along the *z*-axis, generating the reflected (*I*_*r*_) and transmitted (*I*_*t*_) waves.

The superconducting layer’s optical response is governed by the Gorter-Casimir two-fluid model [33]. Below the critical temperature *T*_*c*_, the resistivity of the superconductor is negligible and the superconductor can be treated as lossless. Therefore, the dielectric constant of S can be expressed by,


$$\varepsilon_{s}=n_{s}^{2}=1-\frac{c^{2}}{\omega^{2}\lambda_{{}_{L}}^{2}}$$ (1)

where *λ*_*L*_(*T*_*e*_)=*λ*_*L*_(0)/(1-*f*(*T*_*e*_))^-2^ is the temperature-dependent London penetration depth, defining the characteristic distance a magnetic field can penetrate into the superconductor. *λ*_*L*_(0) is the penetration depth at absolute zero temperature (set to 6100 nm for Hg-1223). The function *f*(*T*_*e*_)=(*T*_*e*_/*T*_*c*_)^4^ is the Gorter-Casimir factor, which models the increase in the density of normal electrons as the temperature *T*_*e*_ approaches the critical temperature *T*_*c*_, leading to an increase in *λ*_*L*_(*T*_*e*_). The critical temperature *T*_*c*_ is pressure-tunable via *T*_*c*_ = 134 + 2.009*P*-0.04194*P*^2^ (GPa) [34]. According to Eq.(1), *n*_*s*_ could be dynamically controlled by *T*_*e*_, *P* and *ω*.

The temperature range discussed in this work is below 200 K. Therefore, the dielectric constant of the layer D varying with the temperature and pressure can be calculated by [35].


$$\varepsilon_{d}=n_{d}^{2}=12.74e^{-1.73\times 10^{-3}P}e^{9.4\times 10^{-5}(T_{e}-75.6)}$$ (2)

Combining Fig 1 with Eqs (1) and (2), the PC’s optical properties are highly sensitive to external conditions.

The transmission and reflection spectra are calculated using the transfer matrix method (TMM) [36], assuming non-absorbing, linear, and homogeneous layers. The relationship between the incident (*E*_0_ and *H*_0_) and output light (*E*_*k+1*_ and *H*_*k+1*_) can be described by the following mathematical model,


$$\left(\;\;\;\begin{array}{c} {}*20cE_{0}\\ H_{0} \end{array}\;\;\;\right)=\prod_{i=1}^{k}M_{i}\left(\;\;\;\begin{array}{c} {}*20cE_{k+1}\\ H_{k+1} \end{array}\;\;\;\right)$$ (3)

where the symbols *E* and *H* respectively denote the electric and magnetic fields. There are totally *k* layers in the PC. The 0-th and *(k + 1)*-th layers are both air. *M*_*i*_ is the equivalent matrix of the *i*-th PC layer, where *i* = 1,2,3,..., *k*. According to the stacking scheme of PC, *M*_*i*_ can be the superconductor (*M*_s_) or dielectric layer (*M*_*d*_). There are four elements in *M*_*i*_ following,


$$M_{i}=M_{s,d}=\left[\;\;\;\begin{array}{cc} {}*20c\cos\varphi_{i} & -\frac{i}{\eta_{i}}\sin\varphi_{i}\\ -i\eta_{i}\sin\varphi_{i} & \cos\varphi_{i} \end{array}\;\;\;\right]$$ (4)

Here, *φ*_*i*_* = 2*π*n*_*i*_*d*_*i*_**cos*θ*_*i*_*/λ* denotes the phase shift accumulated by the wave as it traverses the *i*-th layer. This term is critical as it determines the constructive or destructive interference that gives rise to the photonic bandgap and defect modes. The term *η*_i_ = *n*_*i*_/cos*θ*_*i*_ (*n*_*i*_* = n*_*s,d*_ *and d*_*i*_* = s,d*) is the effective optical admittance for TM light. The main diagonal elements (cos *φ*_*i*_) describe the phase propagation through the layer, while the off-diagonal ones (-*i*sin*φ*_*i*_/*η*_i_ and -**i*η*_i_sin*φ*_*i*_) couple the electric and magnetic fields and describe the reflection and transmission at the interfaces of the layer. The symbol *θ*_*i*_ represents the incident angle of the *i*-th layer. The air boundaries yield *η*_0* *_= *η*_*k+1*_ = (*ε*_*0*_/*μ*_*0*_)^1/2^, where *ε*_*0*_ and *μ*_*0*_ denotes the permittivity and permeability of air. According to the TMM, the transmission *t* and reflection *r* coefficients are derived as,


$$t=\frac{\text{2}}{m11+m12\eta_{0}+m21/\eta_{0}+m22},$$ (5)

$$r=\frac{m11+m12\eta_{0}-m21/\eta_{0}+m22}{m11+m12\eta_{0}+m21/\eta_{0}+m22}.$$ (6)

where *m*_*ij*_ are elements of the total transfer matrix $M=\prod M_{i}$. Consequently, the transmittance and reflectance can be obtained by *T**** ***= tt* and *R*_*** ***_= rr*, respectively [37].

The sensitivity in this work is calculated as *S* = d*y*/d*x*, where *y* is the transmittance *T* or the wavelength *λ*_*c*_ of the DM and *x* is the tunable factors, such as incident angle, ambient temperature, and hydrostatic pressure. The sampled *x-y* pairs are fitted by Least Squares Method to obtain an approximate function. Next, the slope of this function is solved to quantify the sensitivity [38].

## 3. Optical fractal-like transmittance

**Fig 2:**
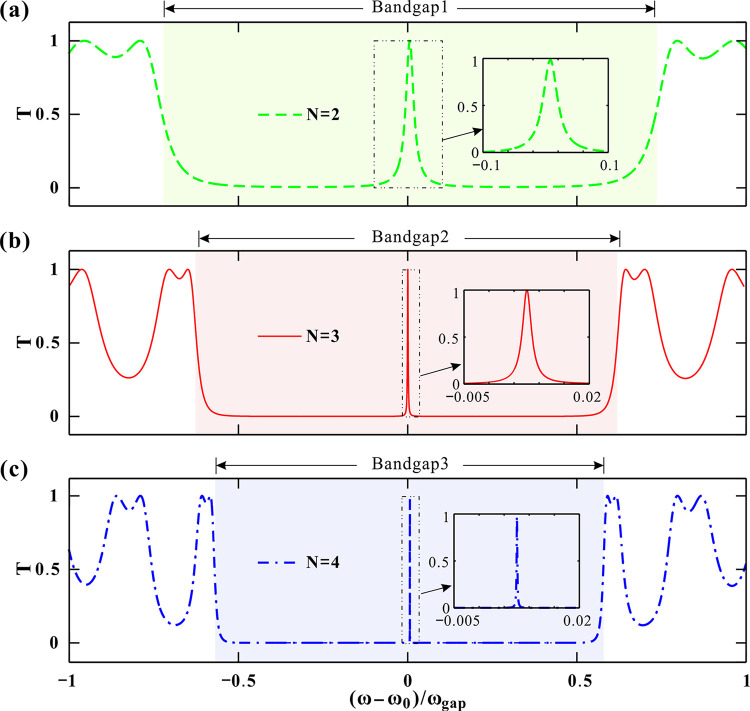
(a-c) Transmittance of periodic superconducting photonic crystal for PC_2_, PC_3_ and PC_4_, respectively. The other parameters are set as *P*=0 GPa, *T*_*e*_=4.2 K and *θ*=0 °.

To elucidate the optical properties of the superconducting PC, we begin by analyzing its transmission spectra. Fig 2 presents the transmittance for period numbers *N* = 2, 3 and 4, plotted against the normalized frequency (*ω* − *ω*_0_*)*/*ω*_gap_, where ω_0_ = 2πc/*λ*_0_ (*λ*_0_ = 1.55 μm) is the center frequency and *ω*_gap_ = 4*ω*_0_arcsin|(*n*_d_-*n*_s)_/(*n*_d_ + *n*_s)_|/π defines the bandgap width [37,39]. Thus, the angular frequency is ω_0_ fixed, while *ω*_gap_ varies with *n*_s_ and *n*_d_. The normalized frequency is utilized to unify these variables to the same frequency scale, making it easier for model construction, simulation and calculation. Previous investigators have found a wide transmission bandgap around the center frequency for periodic PC [37,40]. This conclusion is confirmed again by the transmission spectrum in Fig 2.

A prominent photonic bandgap (Bandgap1) in Fig 2(a), where transmittance *T* drops significantly, is observed around the center frequency. This bandgap is flanked by oscillating transmission peaks. At its center, a sharp, high-transmittance (*T* ≈ 1) DM is present. Here, this DM is not attributed to introducing a foreign material as the defect layer [25,26]. Instead, the two adjacent dielectric (D) layers at the exact center of the (SD)^*N*^(DS)^*N*^ structure constitute a robust and well-defined defect, breaking the translational symmetry of the (SD) lattice and creates a localized state. In Figs 2(b) and 2(c), increasing the number of periods *N* has a profound effect on the spectrum. First, the bandgap compresses. The entire bandgap becomes narrower and more defined. Second, the DM sharpens. The central DM becomes dramatically sharper. The sharpening of the DM with increasing period number N can be quantified by the Full Width at Half Maximum (FWHM). For *N* = 2, 3 and 4, the DM FWHMs are of 3.5 × 10^−2^, 2.5 × 10^−3^ and 2.7 × 10^−4^, respectively. The FWHM decreases by orders of magnitude with larger *N*, while maintaining near-perfect peak transmittance (*T* ≈ 0.99). The quality factors are calculated by Q=*(ω-ω*_c_*)/ω*_*gap*_/FWHM. The center wavelength of the transmission peak almost keeps stable at *(ω-ω*_c_*)/ω*_*gap*_ = 0.075 with varying *N*. Thus, we can achieve the correspondingly increasing Q of 2.14, 30 and 277.8. Third, the edge features. New transmission peak-dip features emerge and multiply at the edges of the bandgap as *N* increases. These properties make the structure highly suitable for an optical band-pass filter. A larger *N* results in a filter with an extremely narrow bandwidth and steeper edges, significantly enhancing its frequency-selection performance [15,29]. Consequently, the period number *N* is a critical parameter for controlling the bandgap width and, most effectively, for tuning the narrow bandwidth of the high-transmittance defect mode.

**Fig 3:**
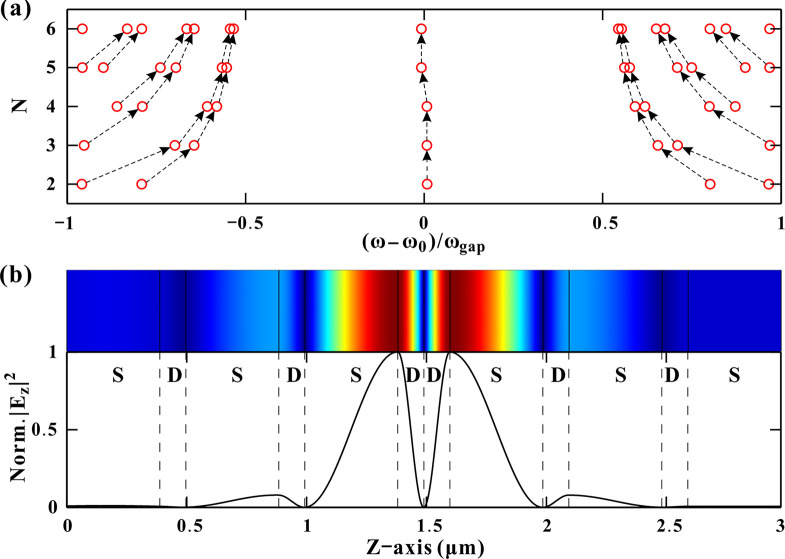
(a) Moving tracks of the transmission peaks as the period number increases. (b) Electric field distribution of the superconducting PC_3_.

To illustrate the peak evolution, Fig 3(a) depicts only the frequency location of the peaks (red circles) according to the transmission spectra in Fig 2. In the center of the bandgap, there is always only one peak for different *N*. As *N* increases, all the peaks on both sides of the bandgap shift towards the center frequency. Each pair of neighboring peaks in a continuous pair move closer to each other. Generally, an optical fractal is a photonic structure or its resultant optical field distribution that exhibits deterministic or statistical self-similarity and scale invariance across multiple levels of spatial or temporal magnification [37]. However, the progressive degeneracy of transmission peaks in our work manifests a discrete scaling of the system. We therefore consider the transmission peak degeneracy as an optical fractal-like phenomenon. The movement of each peak pair is accompanied by peak degeneracy. That is, increasing *N* results not in splitting but in degeneracy of the transmission peaks. The center DM remains singular and stationary, while side peaks exhibit progressive degeneracy. This is quite different from conventional peak splitting [37]. The electric field distributions in Fig 3(b) further confirm this behavior. For N = 3, the inverse TMM and finite-difference time-domain (FDTD) simulations [41] reveal symmetric |*E*_*z*_|^2^ profiles, with a null field at the PC center and field maxima at the S/D interfaces. These interfacial fields stabilize the bandgap and DM, while decaying oscillations correlate with subsidiary peaks. The agreement between TMM and FDTD validates our model.

This phenomenon may be attributed to three factors: the mirror symmetry and field localization, superconducting layer’s optical response and destructive interference of side peaks. First, the PC structure is designed with strict mirror symmetry (SD/DS bilayers), and the defect (two adjacent dielectric layers) is placed at the exact center. This symmetry enforces even-mode dominance for the defect mode (DM), preventing asymmetric splitting. Second, the Gorter-Casimir two-fluid model governs the superconductor’s permittivity (Eq 1). Below *T*_*c*_, the superconductor behaves as a lossless, high-index medium with negligible damping. This results in sharp phase shifts at S/D interfaces and suppression of higher-order resonances. Third, conventional dielectric PCs often exhibit peak splitting due to Fabry-Pérot-like resonances at multiple interfaces. However, in this superconducting PC, the London penetration depth (*λ*_*L*_) of the superconductor introduces an additional length scale that modifies the phase-matching conditions. For larger *N*, the accumulated phase mismatch for off-center frequencies causes destructive interference for side peaks, leaving only the symmetric DM intact. These ultra-narrow DMs are critical assets for high-resolution optical sensing and filtering, as it allows for precise tracking of the resonance wavelength shift, thereby enhancing measurement accuracy and signal-to-noise ratio.

## 4. Optical effect of pressure, temperature and incident angle

**Fig 4:**
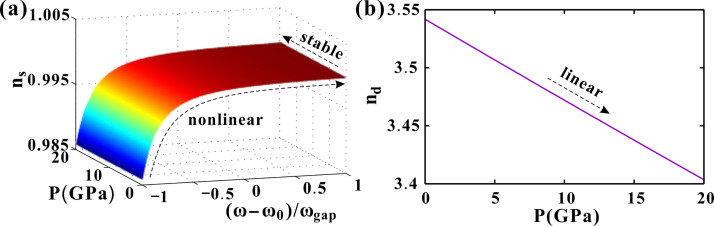
(a) The refractive index *n*_*s*_ of the superconductor varying with the pressure and the frequency. (b) The refractive index *n*_*d*_ of the dielectric layer varying with the pressure. The other parameters are set as *T*_*e*_=4.2 K and *θ*=0 °.

We employ PC_3_ as a representative superconducting PC to systematically investigate its optical response to external stimuli. During the investigation of hydrostatic pressure effects, the ambient temperature and incident angle are fixed at *T*_*e*_ = 4.2 K and *θ* = 0°, respectively. Fig 4(a) illustrates the pressure-dependent refractive index *n*_*s*_ of the superconductor. Below the critical temperature (*T*_*e*_ < *T*_*c*_), *n*_*s*_ remains purely real, exhibiting a gradual increase from 0.985 to 1 across the normalized frequency range, with pressure exerting minimal influence. That is, the main effect on *n*_*s*_ is the frequency. The pressure has a very slight influence on *n*_*s*_. In contrast, Fig 4(b) demonstrates significant pressure sensitivity in the dielectric layer’s refractive index *n*_*d*_, showing a linear decrease from 3.54 to 3.4 as pressure increases from 0 to 20 GPa. The hydrostatic pressure range investigated in this work corresponds to conditions achievable with diamond anvil cell (DAC) technology. Pressures in the 20 GPa range are routinely achieved and accurately calibrated in such setups using the fluorescence shift of standard pressure markers like ruby spheres [42]. This establishes the dielectric layer as the primary pressure-sensing component.

**Fig 5:**
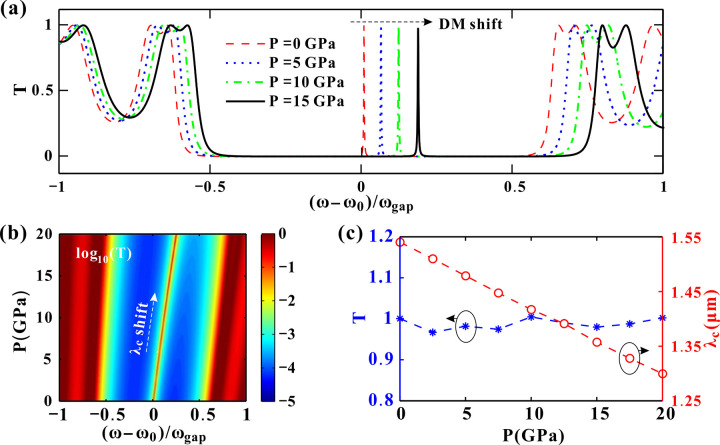
(a) Transmission spectra for different pressures. (b) Logarithm of the transmittance as the pressure changes. (c) Transmittance *T* and wavelength *λ*_*c*_ of the DM varying with the pressure. The other parameters are set as *T*_*e*_=4.2 K and *θ*=0 °.

As shown in Fig 5, the refractive index modifications directly impact the transmission characteristics. While maintaining spectral shape, the entire transmission profile undergoes a blue shift with increasing pressure in Fig 5a. The transmittance of the DM is always close to 1. As *P* increases continuously, the transmittance influenced by pressure is given in Fig 5(b). Logarithmic contrast enhancement reveals a pressure-dependent bandgap expansion. The blue areas correspond to the bandgap. The bandgap width increases smoothly with increasing pressure. The oblique line between the blue areas denotes the DM. Focusing on the DM, Fig 5(c) gives its peak transmittance and the corresponding wavelength *λ*_*c*_. The peak transmittance *T* is quite stable around the average 0.99, fluctuating slightly with the pressure. The peak wavelength decreases proportionally with increasing pressure. As the DM is utilized to develop optical sensors to pressure, this perfect linearity could help eliminate nonlinear errors. More calculation results of the hydrostatic pressure influences are illustrated in S1 Fig.

The primary effect of pressure is to compress the lattice of the dielectric material GaAs, altering its refractive index (*n*_*d*_) via the photoelastic effect governed by Eq. (2). To maintain the resonant condition at the defect (which requires a specific phase delay), the DM wavelength must decrease (blue-shift) to compensate for the reduced phase delay.

**Fig 6:**
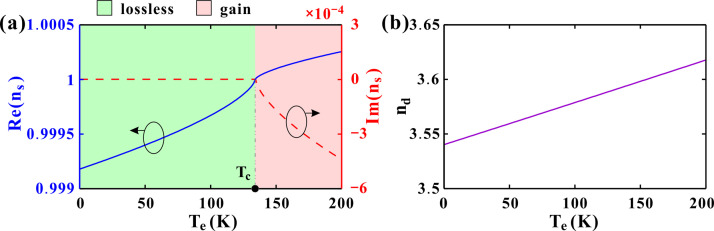
(a) The real and imaginary parts of the superconductor refractive index varying with the temperature. (b) The refractive index of the dielectric layer varying with the temperature. The other parameters are set as *P*=0 GPa and *θ*=0 °.

The effects of temperature, probed at P = 0 GPa and *θ* = 0°, are provided in Fig 6. From a practical standpoint, the operational temperature range of the proposed sensor (4.2 K to 200 K) is readily accessible using standard cryogenic systems. The lower end of this range (4.2 K) is provided by liquid helium bath cryostats or pulse-tube cryocoolers, which are workhorses in superconducting quantum computing and low-temperature physics. Crucially, the upper operational limit of 200 K (−73 °C) is well within the reach of more compact and cost-effective closed-cycle cryocoolers, often referred to as Joule-Thomson or Stirling cryocoolers [43]. These systems are commercially available, highly reliable, and are routinely used to cool infrared detectors and other optoelectronic systems in both laboratory and field-deployable applications, including space-borne instrumentation. The superconductor’s refractive index *n*_*s*_ transitions from purely real (*T*_*e*_ < *T*_*c*_) to complex (*T*_*e*_ > *T*_*c*_), with the real part dominating. As *T*_*e*_ increases from 0 to *T*_*c*_, *n*_*s*_ is real and smoothly increases from 0.9992 to 1. Above *T*_*c*_, a negative imaginary part appears and decreases with the temperature. Meanwhile, the real part continues to increase smoothly from a value of 1. Considering the amplitude, the real part is about 10⁴ times larger than the imaginary one. Fig 6(b) gives the refractive index *n*_*d*_ of the dielectric layer according to Eq.(2). The dielectric’s *n*_*d*_ exhibits a quasi-linear increase from 3.54 (0 K) to 3.62 (200 K).

Temperature affects both constituent materials. Low-temperature operation is well-established and actively pursued in the field, as evidenced by recent high-impact publications [14,15,44]. the core functionality of our device hinges on the exceptional electro-optical properties of the superconducting layer when it is in the superconducting state, which occurs below its critical temperature (*T*_*c*_). For the superconductor refractive index in Fig. 6(a), the real part Re(*n*_*s*_) increases smoothly with temperature as *T*_*e*_ < *T*_*c*_. As *T*_*e*_ continues to increase, Hg-1223 transitions to a normal state with complex permittivity, but Re(*n*_*s*_) continues to increase. In Fig. 6(b), the dielectric GaAs exhibits quasi-linearly increasing *n*_*d*_ with temperature due to thermo-optic effect and lattice expansion. An increase in either *n*_*s*_ or *n*_*d*_ lengthens the optical path in the layers. To maintain the resonance condition at the defect, the DM wavelength must increase (red-shift).

**Fig 7:**
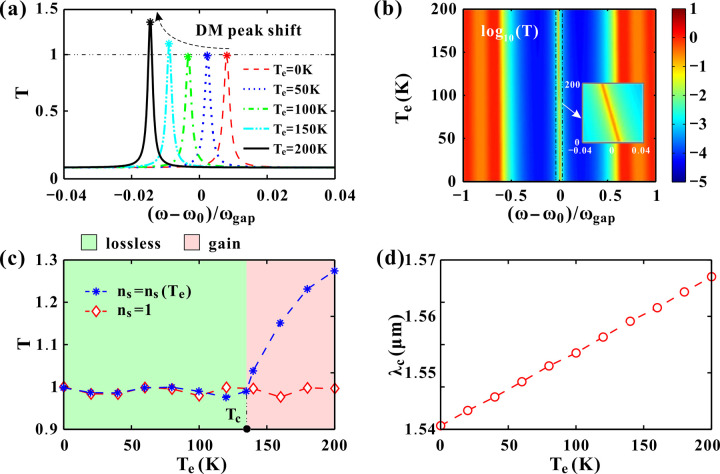
(a) Transmission spectrum around the DM for different temperatures. (b) Logarithm of the transmittance as the temperature changes. (c) Transmittance comparison of the DM varying with the temperature between *n*_*s*_ = *n*_*s*_(*T*_*e*_) and *n*_*s*_ = 1. (d) Wavelength of the center peak varying with the temperature. The other parameters are set as *P*=0 GPa and *θ*=0 °.

The above variations of *n*_*s*_ and *n*_*d*_ induce a temperature-dependent red shift in the DM illustrated in Fig 7(a). The center DM varies significantly with the temperature. As *T*_*e*_ increases, the center DM shifts continuously to lower frequency. In the meantime, the peak bandwidth almost remains the same. For *T*_*e*_ = 0, 50 and 100K, the peak transmittance are all close to 1. As *T*_*e*_ ranges form 150K to 200K, the transmittance exceeds 1 at elevated temperatures. Higher temperatures lead to larger transmittance of the DM. Fig 7(b) gives the transmittance varying with the temperature and frequency. The bandwidths of the bandgap (the blue regions) and DM (the yellow line) almost remain the same. The entire transmission bandgap shifts slightly, demonstrating a smooth redshift in frequency with increasing temperature. When magnifying the center DM in the sub-box, the oblique trajectory of the DM can be clearly observed. To make a comparison between the temperature-sensitive superconductor *n*_*s*_(*T*_*e*_) and the temperature-independent material (*n*_*s*_ = 1), Fig 7(c) provides the transmittance of the DM at ω = ω_0_ for different temperatures. For a fixed *n*_*s*_ = 1, the peak transmittance slightly fluctuates below *T* = 1 as the temperature increases from 0 K to 200 K. This fluctuation depends on the other constituent layer GaAs according to Eq.(2). For our selected superconductor Hg-1223, the peak transmittance also fluctuates below 1 at temperatures below the critical temperature *T*_*c*_ = 134 K. Nonetheless, the transmittance increases sharply and monotonously as *T*_*e*_ > *T*_*c*_. Consequently, the transmitted light intensity is enhanced, which facilitates its detection. Fig 7(d) gives the wavelength of the DM for different temperatures. The approximately equidistant center peaks in Fig 7(a) leads to the good linearity of the relationship between *λ*_*c*_ and *T*_*e*_. Notably, the DM wavelength maintains a linear temperature dependence, while the transmittance shows an abrupt enhancement above *T*_*c*_, suggesting dual-mode thermal sensing capability. This dual-response—linear wavelength shift and abrupt transmittance jump—provides a unique dual-mode thermal sensing capability, useful both for cryogenic thermometry and for precisely detecting the superconducting transition point itself.

As illustrated in Fig 6(a), Loss is rigorously accounted for in the superconducting state via the Gorter-Casimir model, which, below *T*_*c*_, results in a purely real refractive index, justifying the “lossless” assumption for the operational regime. However, loss (and indeed, gain) is explicitly included and becomes a central feature when the superconductor transitions to its normal state. As *T*_*e*_ continuous to increase beyond *T*_*c*_, Im(*n*_*s*_) is negative and |Im(*n*_*s*_)| increases. For example, *n*_*s*_ = 1.0001–2 × 10^-4^*i* at *T*_*e*_ = 150 K*.* Here, the negative imaginary part denotes the introduction of gain [39]. Under this specific conditions, transmittance can be greater than 1. This indicates that the system is no longer passive and lossless but is instead exhibiting optical gain, meaning it is amplifying the light. Consequently, the non-zero negative imaginary part of the refractive index represents gain, resulting in *T* > 1 in Fig 7(c). The most significant physical insight is the abrupt and substantial increase in transmittance occurring precisely at *T*_*c*_. This sharp transition provides a novel and highly sensitive mechanism for device functionality. It enables a dual-mode thermal sensing capability, where the wavelength offers continuous temperature readout below *T*_*c*_, and the transmittance provides a discrete, high-contrast signal for pinpointing the superconducting transition itself, useful for optical switching or critical-temperature monitoring. More calculation results of the temperature influences are provided in S2 Fig.

**Fig 8:**
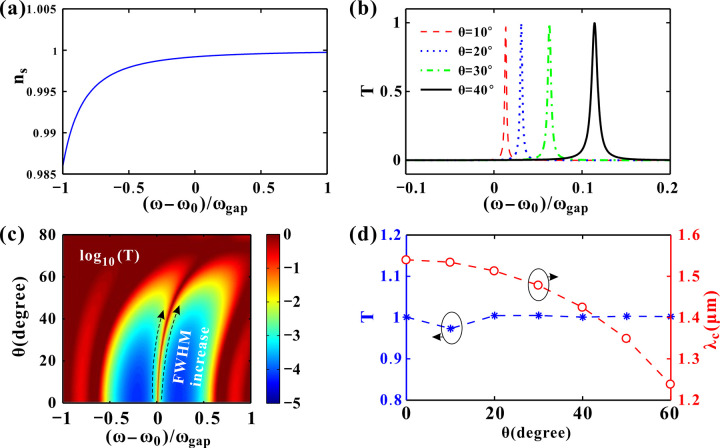
(a) The refractive index of the superconductor varying with the frequency. (b) Transmission spectra for different incident angles. (c) Logarithm of the transmittance as the incident angle changes. (d) Transmittance and wavelength of the DM varying with the incident angle. The other parameters are set as *P*=0 GPa and *T*_*e*_=4.2 K.

The incident angle is another factor to influence the transmittance of PCs. The modulation of the incident angle *θ* in a practical sensor system can be realized using a standard goniometer-based optical setup. The superconducting photonic crystal chip would be mounted on a rotation stage at the center of the goniometer, allowing for precise control of *θ* [3,21]. As illustrated in Fig 8(a), the refractive index of superconductor *n*_*s*_ is merely related to the frequency as *P* = 0 GPa and *T*_*e*_ = 4.2 K. This *n*_*s*_ curve is approximately the same as the cross-section shown in Fig 4(a). Fig 8(b) gives the transmission spectra around the DM for different incident angles. As *θ* increases from 10° to 40°, the DM shifts to higher frequency and its bandwidth becomes wider. Meanwhile, all the transmittance of the peaks are close to 1. Fig 8(c) demonstrates the continuous variation of the transmittance. The overall transmittance pattern exhibits a bifurcated structure that evolves with angle. As *θ* increases from 0° to 20°, the transmittance almost remains. For larger *θ*, the transmission spectrum shifts to higher frequency apparently. The center peak expands with larger FWHM and both the the bandgap and DM contours become smooth. As *θ* > 75°, the bandgap disappears. The transmittance and wavelength of the center peak are illustrated in Fig 8(d). The transmittance almost remains close to 1 for varying incident angles. That is, the transmitted light intensity of the center peak is always strong enough to be detected. On the other hand, the corresponding wavelength of the center peak *λ*_*c*_ is quite sensitive to the incident angle *θ*. Remarkably, the DM transmittance remains 1 across all angles, while *λ*_*c*_ decreases nonlinearly from 1.55 μm to 1.24 μm, suggesting potential for use in angular photonic sensors. More calculation results of the incident angle influences are given in S3 Fig. The highly sensitive and nonlinear angular response is promising for developing ultra-precision angular alignment systems and angle-to-wavelength encoders in compact photonic circuits.

Incident angle is a purely geometric effect related to the path of light through the layered structure, governed by Snell’s law [45] and the phase condition for resonance. For a plane wave incident at an angle *θ*, the effective path length it travels in a layer of thickness *d* is *d*/cos*θ*_*i*_, where *θ*_*i*_ is the refracted angle inside the layer. The phase shift accumulated in a layer is crucial for defining the photonic bandgap and the DM. It can be calculated by *φ*_*i*_=(2π/*λ*) *nd*_*i*_ cos*θ*_*i*_ (Eq. 4). As *θ* increases, cos*θ*_*i*_ decreases. To maintain the same phase shift *φ*_*i*_ required for resonance (e.g., the quarter-wave condition *φ*_*i*_ = π/2), the wavelength *λ* must decrease.

**Fig 9:**
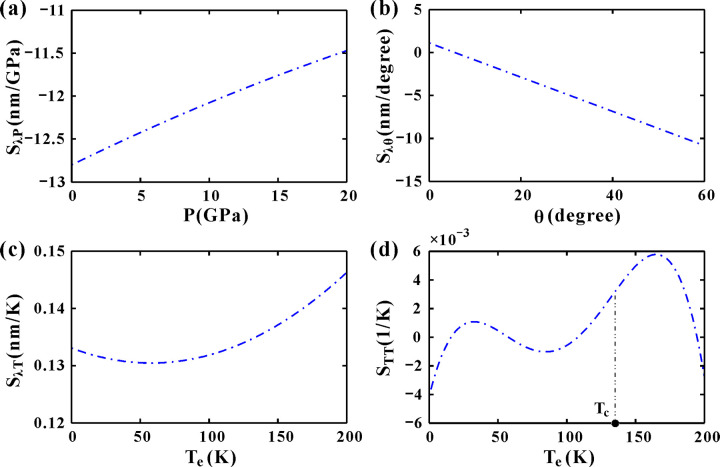
(a-c) The sensitivity of the wavelength at the DM varying with the pressure (*θ*=0° and *T*_*e*_=4.2 K), incident angle (*P*=0 GPa and *T*_*e*_=4.2 K) and the temperature (*P*=0 GPa and *θ*=0°), respectively. (d) The sensitivity of the transmittance varying with the temperature (*P*=0 GPa and *θ*=0°).

Combining Figs 5c, 7c, 7d and 8d, the wavelength and transmittance sensitivity of the DM are illustrated in Fig 9. The effect of only hydrostatic pressure is considered in Fig 9a. The wavelength sensitivity *S*_*λP*_ is always negative by exerting different pressures. That is, high pressure corresponds to short incident wavelength. Hydrostatic pressure compresses the lattice, altering the dielectric constant (*n*_*d*_) of GaAs via the photoelastic effect (Eq. 2). The superconductor’s refractive index (*n*_*s*_) is less pressure-sensitive (Fig 4a), making *n*_*d*_ the dominant factor. Increased pressure reduces *n*_*d*_ (Fig 4b), shrinking the optical path length (*n*_*d*_·d) in dielectric layers. This blue-shifts the DM wavelength (*λ*_*c*_) to compensate for the reduced phase delay, maintaining resonance at the defect. Consequently, negative *S*_*λP*_ is obtained. Meanwhile, |*S*_*λP*_| decreases by exerting higher pressures. This trend is reversed when modulating the incident angle *θ*, as shown in Fig 9b. While increasing *θ*, the wavelength sensitivity |*S*_*λθ*_| increases as well. For TM-polarized light, the effective optical admittance *η*_*i*_* = n*_*i*_/cos*θ*_*i*_ (Eq. 4) decreases with larger *θ*, modifying the phase condition for resonance. Meanwhile, larger *θ* reduces the effective optical thickness (*n*_*i*_*d*_*i*_cos*θ*_*i*_), requiring a shorter *λ*_*c*_ to satisfy the quarter-wave condition. This makes *S*_*λθ*_ negative. The cos*θ*_*i*_ term in *η*_*i*_ introduces a geometric nonlinearity. This amplifies the wavelength shift at grazing angles and results in |*S*_*λθ*_|growth with *θ*.

In Fig 9(c), the wavelength sensitivity *S*_*λT*_ is always positive as *T*_*e*_ > 0. The incident wavelength increases with the temperature. Higher temperatures yield higher sensitivity. As the temperature *T*_*e*_ is below *T*_*c*_, The superconductor’s *n*_*s*_ increases with *T*_*e*_ (Fig 6a), while GaAs’s *n*_*d*_ expands quasi-linearly (Fig 6b). For *T*_*e*_ > *T*_*c*_, *n*_*s*_ becomes complex, but its real part still grows, dominating the response. Thermal expansion and increased *n*_*s*_ and *n*_*d*_ lengthen the optical path, requiring a redshift in *λ*_*c*_ to maintain resonance. This makes *S*_*λT*_ positive. Temperature affects both *n*_*s*_ and *n*_*d*_ additively, partially compensating for each other’s impact on the phase condition. Fig 9(d) gives the transmission sensitivity *S*_*TT*_ of the center peak. The relationship between *S*_*TT*_ and *T*_*e*_ is characterized by a two-peak (bimodal) structure. For the left hump, the superconductor is lossless (Im(*n*_*s*_)=0 and T ≈ 1 (Fig 7c). Weak sensitivity stems from small changes in *n*_*d*_. The steepest rising edge of the right hump corresponds to the critical temperature *T*_*c*_. The transition to the normal state introduces dissipation(Im(*n*_*s*_)≠0), but the real part of *n*_*s*_ grows sharply (Fig 6a), creating a Fano-like resonance, which abruptly enhances the transmittance due to interference between lossy and coherent pathways. The right hump with high temperature (*T*_*e*_ > *T*_*c*_) has high sensitivities. The system behaves like a metal-dielectric PC, where increasing *T*_*e*_ further raises Re(*n*_*s*_), sustaining high transmittance (Fig 7c).

The sensitivity analysis presented in Fig 9 quantifies the following effects. First, the pressure sensitivity *S*_*λP*_ shows negative trends that decrease with pressure. Second, the angular sensitivity |*S*_*λθ*_| increases monotonically with the angle. Therefore, the optimal operating point for angular sensing is at the largest practical incident angle *θ* before the bandgap collapses (approximately *θ* > 75°), where the sensitivity is highest. Third, Thermal wavelength sensitivity *S*_*λT*_ is positive but an order of magnitude weaker. *S*_*λT*_ is higher at elevated temperatures (e.g., *T*_*e*_ > 150 K). Fourth, Thermal transmittance sensitivity *S*_*TT*_ peaks near *T*_*c*_. The demonstrated linear responses and high sensitivities (about 10^−3^ order) positions this superconducting PC as an exceptional platform for pressure and angle sensing applications. Combining the pressure, temperature and incident angle, we achieve a high pressure sensitivity of up to 128 nm/Gpa as *P* = 0 GPa, *T*_*e*_ = 200 K and *θ* = 60°.

Table 1 presents the pressure sensor comparison with recent investigations [44,46,47]. The Silicon PC with periodic Si/Air pairs is the most sensitive to hydrostatic pressure. However, the air layer may lead to serious deformation with high pressures [47]. Our proposed superconducting PC with mirror symmetry has the second largest sensitivity, while maintaining a certain rigidity to prevent deformation. The achieved high pressure sensitivity of 128 nm/GPa, coupled with the robust solid-state structure, positions this PC as an excellent platform for high-pressure monitoring in cryogenic environments, such as around superconducting magnets or in high-pressure material physics setups. Combining Fig 9 with Table 1, it can be concluded that the mirror-symmetric HTSC PC presented in our work provides a specialized platform that excels in high-sensitivity, multi-parameter optical sensing by offering a uniquely sharp, stable, and linearly responsive defect mode. The pursuit of sensors that are both highly sensitive and robust to structural imperfections is a key goal in photonics. Recent advances in topological photonics, for instance, have demonstrated systems where edge states are protected against internal disorder while remaining exquisitely sensitive to specific external perturbations [48]. While the origin of robustness in our symmetric PC is distinct—arising from a deterministic defect rather than a topological invariant—it similarly achieves a favorable synergy of high sensitivity to external stimuli and inherent stability, offering a complementary pathway to high-performance sensing. This is a combination of features not fully realized in the other HTSC PC architectures [22,26,33].

**Table 1:** Pressure sensor comparison with recent investigations.

Pressure sensor	Structure	Sensitivity	Year
Ref. [44]	Superconductor PC with GaAs defect	1.75 nm/GPa	2022
Ref.[46]	PC with Al_2_O_3_ defect	72 nm/GPa	2023
Ref. [47]	Silicon PC with periodic Si/Air	350 nm/GPa	2023
Ours	Superconducting PC with mirror symmetry	128 nm/GPa	2025

## 5. Conclusions

In summary, we have demonstrated a novel superconducting photonic crystal (PC) architecture featuring mirror-symmetric SD/DS periodic bilayers with intentionally introduced dielectric defect layers. The structure exhibits several remarkable optical characteristics under TM-polarized illumination at the design wavelength of 1.55 μm. A pronounced photonic bandgap emerges, containing a sharply defined defect mode (DM) that effectively splits the bandgap into two distinct regions. Instead of the splitting optical fractal, the transmission peaks degenerate as the periodic number *N* increases. When combined with this fractal-like behavior, external stimuli produce highly predictable optical responses. Hydrostatic pressure induces linear blue-shifting of the DM wavelength while maintaining near-unity transmittance. Increasing the temperature causes monotonic red-shifting, with a distinct transmittance transition at *T*_*c*_ (remaining stable below *T*_*c*_ but increasing dramatically above it). Varying the incident angle variation generates parabolic wavelength modulation without compromising transmittance. The system shows exceptional sensitivity profiles, with optimal performance achieved at lower pressures, elevated temperatures, and larger incidence angles. The practicality of our superconducting photonic crystal sensor is anchored in three key aspects: robust solid-state design, compatibility with existing cryogenic infrastructure, and multi-parameter sensing capability within a single, compact platform. The unique integration of superconducting electrodynamic properties with photonic bandgap engineering establishes a novel paradigm for developing ultra-sensitive, multifunctional optical sensors. Regarding integration with scalable systems, the proposed sensor aligns with on-chip integration within cryogenic electronic systems and general cryogenic monitoring.

## Supporting information

S1 Fig (a) The refractive index *n*_*s*_ of the superconductor varying with the frequency. **(b) The refractive index *n***_***d***_ **of the dielectric layer varying with the pressure. (c) Transmission spectra for different pressures. (d) Transmission spectrum around the DM for different pressures (*P* = 0, 2.5, 5,…, 17.5, 20 GPa).** The other parameters are set as *T*_*e*_ = 4.2 K and *θ* = 0 °.
(TIF)

S2 Fig (a) Real and (b) imaginary parts of the superconductor refractive index varying with the temperature. **The refractive index of the dielectric layer varying with the temperature. (c) Transmission spectrum around the DM for different pressures (*T***_***e***_** = 0, 20, 40,…, 180, 200 K). (d) Transmission spectrum around the DM for different temperatures. (e) The refractive index *n***_***s***_ **of the superconductor varying with the frequency and normalized frequency.** The other parameters are set as *P* = 0 GPa and *θ* = 0°.
(TIF)

S3 Fig (a) Transmission spectrum around the DM for different incident angles (*θ* = 0°, 10°, 20°,…, 50°, 60°). **(b) Transmission spectrum around the DM for different incident angles. (c) Transmission spectrum varying with the incident angle and normalized frequency.** The other parameters are set as *T*_*e*_ = 4.2 K and *P* = 0 GPa.
(TIF)

S1 File DM tunability via Pressure (P). The other parameters are set as *T*_*e*_ = 4.2 K and *θ* = 0 °.
(ZIP)

S2 File DM tunability via temperature (*T*_*e*_). The other parameters are set as *P* = 0 GPa and *θ* = 0°.
(ZIP)

S3 File DM tunability via incident angle (*θ*). The other parameters are set as *T*_*e*_ = 4.2 K and *P* = 0 GPa.
(ZIP)
