# Supplementary figures and images for: Tunable narrow-and-sharp defect modes and transmission peak degeneracy in periodic superconducting photonic crystals
Source: PLoS One. 2026 Jan 23;21(1):e0341241. doi: 10.1371/journal.pone.0341241 (PMC12829875; doi:10.1371/journal.pone.0341241)

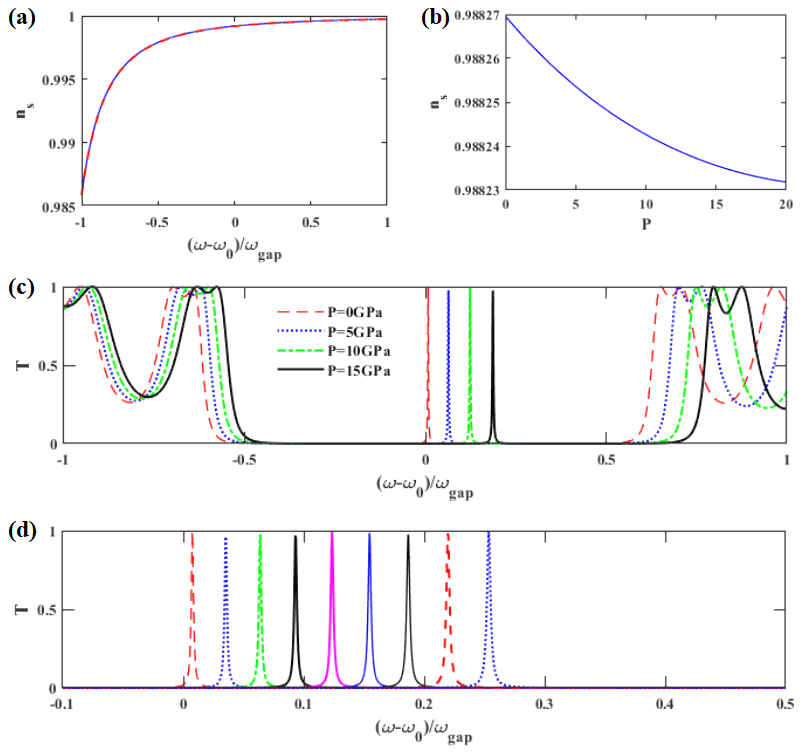

Supplement: S1 Fig — (b) The refractive index nd of the dielectric layer varying with the pressure. (c) Transmission spectra for different pressures. (d) Transmission spectrum around the DM for different pressures (P = 0, 2.5, 5,…, 17.5, 20 GPa). The other parameters are set as Te = 4.2 K and θ = 0 °. (TIF) [file pone.0341241.s001.tif]

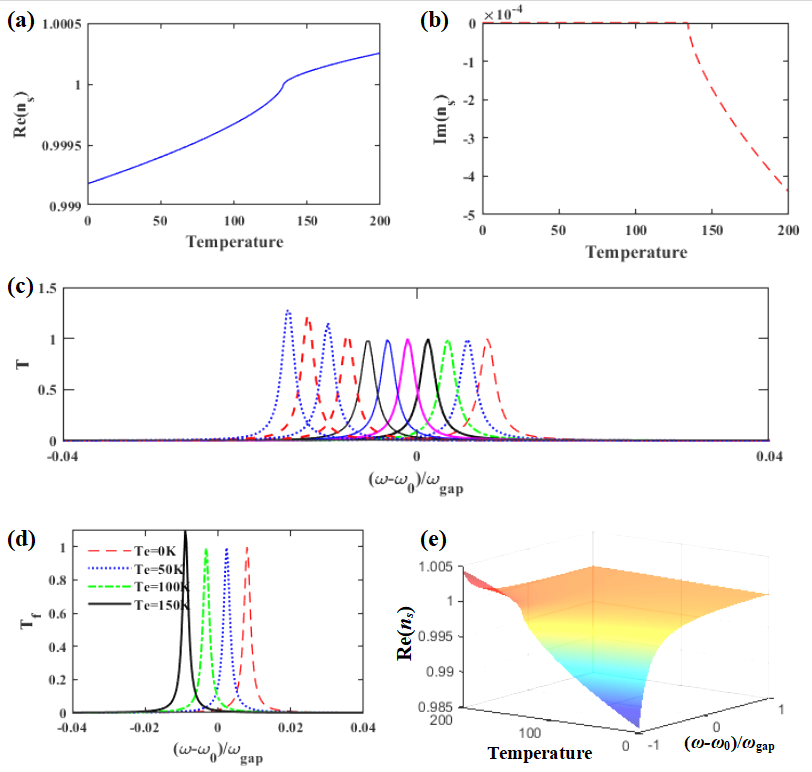

Supplement: S2 Fig — The refractive index of the dielectric layer varying with the temperature. (c) Transmission spectrum around the DM for different pressures (Te = 0, 20, 40,…, 180, 200 K). (d) Transmission spectrum around the DM for different temperatures. (e) The refractive index ns of the superconductor varying with the frequency and normalized frequency. The other parameters are set as P = 0 GPa and θ = 0°. (TIF) [file pone.0341241.s002.tif]

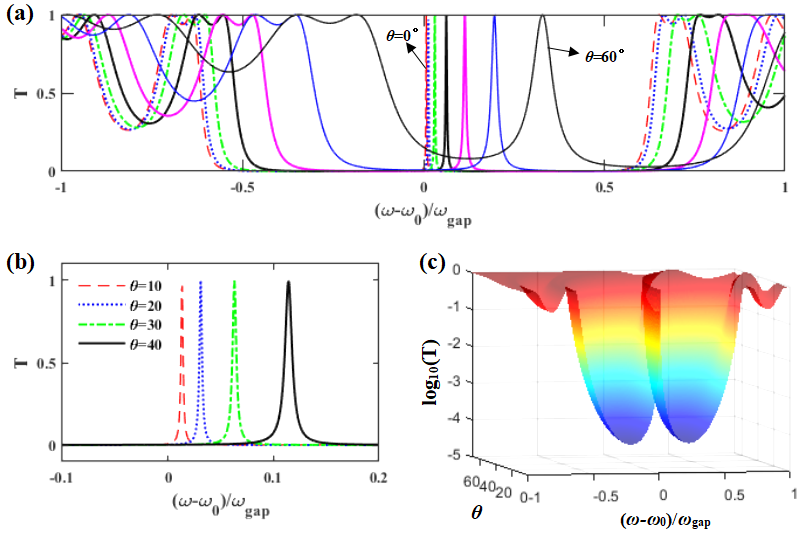

Supplement: S3 Fig — (b) Transmission spectrum around the DM for different incident angles. (c) Transmission spectrum varying with the incident angle and normalized frequency. The other parameters are set as Te = 4.2 K and P = 0 GPa. (TIF) [file pone.0341241.s003.tif]
